# Using Zoo Welfare Assessments to Identify Common Issues in Developing Country Zoos

**DOI:** 10.3390/ani10112101

**Published:** 2020-11-12

**Authors:** Samantha J. Ward, Ellen Williams, Georgina Groves, Simon Marsh, David Morgan

**Affiliations:** 1 School of Animal, Rural and Environmental Sciences, Nottingham Trent University, Nottingham NG25 0QF, UK; ellen.williams@ntu.ac.uk; 2 Wild Welfare, West Sussex RH10 1HT, UK; georgina@wildwelfare.org (G.G.); simon@wildwelfare.org (S.M.); dave@wildwelfare.org (D.M.)

**Keywords:** welfare, audit, welfare assessment, animal management, animal care, zoo, wildlife

## Abstract

**Simple Summary:**

Zoo animal welfare is a high priority for many institutions. Modern zoos try to ensure that animals are housed and managed at high standards, using animal welfare assessments based on scientific evidence-based practices. However, animal welfare standards for developing country zoos may not be as high, as the most up-to-date knowledge may not be available or understood. The aim of this research was to investigate if there were common welfare concerns associated with zoo animal provision across different developing country zoos. Zoo welfare audits were completed at 11 zoos in seven developing countries (Brazil, Egypt, Libya, Indonesia, Thailand, Malaysia and Vietnam). The results suggest that animal behaviour, animals’ positive mental states and human health and safety were areas that needed support. These common themes were likely due to a lack of knowledge and understanding that may be linked to historical and cultural differences. This research has helped to inform future intervention strategies for improving developing country zoo animal welfare.

**Abstract:**

Zoo animal welfare is a high priority for many institutions worldwide, with modern zoos now ensuring that animals are housed and cared for to the highest standards. However, in countries where this knowledge is not as available or understood, standards may be lower. The aim of this research was to investigate if there were common zoo welfare concerns across developing country zoos. Wild Welfare is a charity working globally to improve welfare for zoo animals and has an independent welfare audit that is carried out before any intervention occurs. The Wild Welfare Audit, consisting of 110 questions, covering nine topics, was completed at 11 zoos in seven developing countries (Brazil, Egypt, Libya, Indonesia, Thailand, Malaysia and Vietnam) following a Likert scale score (1–3). A principal component analysis was also performed to evaluate the audit questions. The results suggest that common areas of concern were animal behaviour, positive animal mental states and human health and safety. These themes were likely due to the lack knowledge and understanding that may be linked to historical and cultural differences. This research has helped to revise the welfare audit as well as inform future intervention strategies for improving developing country zoo animal welfare.

## 1. Introduction

The exact number of zoos and similar public animal facilities around the world is unknown, but estimates are around 10,000 [1]. Some of these zoos will be members of professional zoo and aquarium associations that occur at national, regional and international levels, such as the Brazilian Association of Zoos and Aquariums (AZAB), the European Association of Zoos and Aquaria (EAZA) and the World Association of Zoos and Aquaria (WAZA). Most member institutions within accredited-run associations show a considered degree of care and management of zoo animals, and this is one of the primary uniting factors of their accredited members, who are striving to achieve higher standards of welfare with increasing advances in zoo animal welfare knowledge [2]. However, the number of captive wild animal facilities that fall outside the ethical oversight of a professional zoo association is concerning [3]. These same institutions often lack institutional governance and appropriate leadership pertaining to short- and long-term animal management that impacts animal welfare.

“Animal welfare” has many definitions, as our knowledge on the subject has advanced over the years. It includes supporting an animal’s psychological and physiological needs [4] and more recently focuses on an animal’s ability to feel both positive and negative emotions [5,6]. Zoo animal welfare is one of the key components in modern zoos achieving their aims (conservation, education, research and recreation). For example, with low welfare standards, an animal’s reproductive success decreases [7,8]. Godinez et al. [9] also found that increases in animal stereotypies decreased visitor enjoyment, reducing the zoo’s opportunities to contribute towards both in situ and ex situ conservation strategies [10]. Additionally, with reduced animal welfare, visitors may be less likely to support the zoo financially or make a return visit [11,12], suggesting that visitors may not have a positive experience if animal welfare is low. Essentially, a successful zoo is one where animal welfare is high and is maintained through appropriate assessment and evaluation.

Measuring zoo animal welfare involves a multi-faceted approach to ensure the effective evaluation of the animals’ psychological and physiological needs. Techniques to measure zoo animal welfare typically include measuring the animal’s behaviour and physiology such as behavioural observations [13], heart rate variability [14] and cortisol [15] (animal-based parameters), as well as resource-based parameters such as the animal’s environmental conditions or provisions [13]. Both animal- and resource-based parameters fall into components formulated originally as the Five Freedoms, referring to what provisions an animal needs for good welfare. With a more modern take on the Five Freedoms, Mellor and Beausoleil [16] devised the Five Domains, which have since been updated to now include human–animal interactions [17]. This places more emphasis on animal feelings and refers to what provisions are needed for a positive valence of welfare.

Currently there are few species-specific tools to measure animal welfare in zoos. Across the world, two of the species which have received the most input in recent years are African and Asian elephants, with a number of researchers seeking to develop systematic methods for welfare assessment [18,19,20,21]. However, this stems from widespread and publicized concern about their welfare [22,23,24,25]. The most recent behavioural welfare assessment tool for elephants was developed as a response to concerns over elephant welfare in UK zoos [26] and was designed for use by elephant keepers to provide quick and reliable behavioural monitoring for evaluation over time [21]. Other species-specific tools such as the one developed for dolphins (*Tursiops truncatus*) [27] or Dorcas Gazelles (*Gazella dorcus*) [28] were based on farm animal welfare protocols such as the Welfare Quality Network^®^ [29], where a number of species-specific tools to measure welfare have been developed and which highlights the need to share techniques across facilities [30]. With only these few specific frameworks available, reliably assessing species-specific animal welfare in zoos is challenging and time consuming. This usually results in zoos utilizing broad-spectrum welfare tools [31], not monitoring welfare on a regular basis, or monitoring through more traditional measures that evaluate the mitigation of poor welfare, rather than the promotion of positive welfare.

Wild Welfare [32] is a charity working globally to improve welfare for wild animals living in captivity and has a welfare auditing system, based on the Five Domains, that is carried out before any intervention occurs [33]. Utilizing their pre-intervention audit, the aim of this research was to investigate if there were common welfare concerns associated with zoo animal provision across a number of developing country zoos, identifying the possible reasons behind these and considering strategies which can be utilized for improving zoo animal welfare. In addition, we aimed to consolidate the Wild Welfare Audit for future assessments.

## 2. Materials and Methods

### 2.1. Data Collection

The Wild Welfare (WW) Audit (see Supplementary Materials File 2) is a multi-species audit that is scored by one of two trained WW staff, independent of the zoos that they assess. The auditors are experienced members of the WW team with extensive international experience in captive animal management and welfare. The primary purpose of the audit is providing an aide memoire for auditors, auditing against the WW Core Fundamentals Standard [34], rather than use as a research tool. The audit comprises 110 questions relating to (i) nutritional provision (nutrition), (ii) the environment in which the animals are housed (environment), (iii) animal health, (iv) animal behaviour (behaviour), (v) the perceived mental state of the animals (mental health), (vi) the standard of animal record keeping (record keeping), (vii) staff health and safety (health and safety), (viii) information relating to the personnel working at the zoo (personnel) and (ix) other (containing questions pertaining to financial support and waste disposal). These topics were included in the audit to enable an evaluation of the whole collection and therefore practices that can impact the welfare of an animal at any one time. For each zoo, the questions received a Likert scale score of unacceptable (1), questionable (2) or acceptable (3) according to modern zoo welfare requirements [23]. Zoos might also score “Not applicable” or “not provided access/information”, but these responses were not taken into account during analysis. The exact same audits were conducted between 2012 and 2018 in 11 zoos (anonymized) in seven countries (Brazil, Egypt, Libya, Indonesia, Thailand, Malaysia and Vietnam).

### 2.2. Ethics Statement

All the research protocols were approved by the Nottingham Trent University, School of Animal, Rural and Environmental Sciences School Ethics Group (reference number ARE192038). The welfare audits were carried out with the permission of the participating institutions. All the zoos were anonymized and are unidentifiable at the zoo and country levels.

### 2.3. Data Analysis

Descriptive statistics pertaining to the nine sections of the questionnaire were gathered using Microsoft Excel. All other statistical analyses were undertaken using R Version 1.2.1335 [35] and SPSS Version 24 [36]. For the audit evaluation, the Likert data created from the questionnaire were converted into polychoric correlation matrices to enable a Principal Component Analysis (PCA) to be undertaken on the data. The polychoric correlation matrices were calculated in R using the packages “Psych” and “Factoextra”. A PCA was conducted to reduce the questionnaire into thematic components. The component solution was rotated using varimax rotation, and components with eigenvalues >1 were extracted. The sampling adequacy was assessed using Bartlett’s test of sphericity, which was performed in R using the package “Parameters”. The PCA was undertaken in SPSS. Adjectives with salient loadings (>0.6) on more than one component were assigned to the components to which they had higher loadings. Questions which loaded negatively onto the component were reverse scored (1 minus the polychoric correlation value). Cronbach’s alpha was used to detect the internal consistency within components.

## 3. Results

### 3.1. Audit Topics

**Figure 1:**
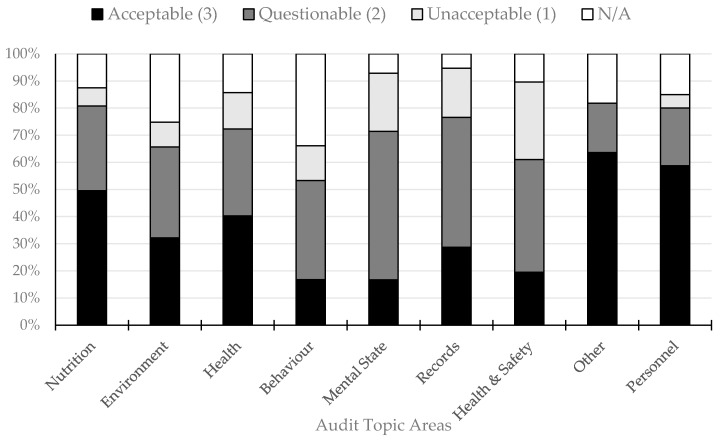
Overview of areas scored as having acceptable (scored 3), questionable (scored 2), unacceptable (scored 1) and N/A (not assessed or not available to assess) practices across all zoos assessed in the Wild Welfare Audit.

For all zoos combined, there were only two topic areas that scored above 50% within the acceptable range (“other”: 63% and “personnel”: 59%; Figure 1). The areas with the lowest frequencies of a score of “acceptable” across the zoos were “behaviour” (17%), “mental state” (17%) and “health and safety” (20%). The area with the highest frequency of a score of “acceptable” was “other” (64%). The numbers of “questionable” scores were similar across all the topics apart from “mental state”, which had the highest number of “questionable” scores (55%; Figure 1). “Other” was the only topic that did not receive any unacceptable score across the zoos, with the remainder of the topics scoring between 5 and 29% of the questions as “unacceptable”. There was a high number of “not assessed/not available to assess” questions for “behaviour” (34%; randomly spread across the questions), with the remaining topic percentages ranging between 5 and 25% for this score. A full breakdown of the areas of acceptable, questionable and unacceptable practices across all the zoos is provided in Supplementary Materials File 1 (Boxes S1–S9).

#### 3.1.1. Nutritional Provision

Zoos scored relatively highly across this area of questions (Box S1). All the zoos were rated as “acceptable” for “sourcing food from a reputable supplier”, “providing food in a manner that is safe for both animals and staff” and “providing appropriate quality foodstuffs”. The modal score of 3 (acceptable) was awarded for a number of areas of practice, including the “general body condition of animals”, “access to clean water”, “food from reputable suppliers”, “correct feed storage” and “dietary review”. There were, however, some areas of concern (where some zoos achieved a score of “unacceptable”), including “animal body weight”, “access to clean water” and “dietary review”. All the zoos scored 1 (unacceptable) for the “provision of live vertebrates as food”. Areas of questionable practice (modal score of 2) included the provision of dietary supplementation and provision of food/drink in a way which met the biological and behavioural needs of the animal.

#### 3.1.2. Housing Environment

The zoos again scored relatively well across this area of questioning (Box S2). All the zoos were rated as “acceptable” for “effectiveness in containing animals within their enclosures” and “facilities for crating and transporting animals if required”. The modal score of 3 (acceptable) was awarded for several areas of practice, including the “cleanliness and maintenance of enclosures”, “safety of personnel”, “enclosure servicing” and “routine veterinary examinations prior to transport”. Areas which were considered “acceptable” across all of the study zoos were the presence of gates/doors which were effective in containing animals, and facilities for crating and transporting animals. There were some areas of concern where some zoos were scored as unacceptable: “overburdened carrying capacity”, “a lack of assessment of animals kept in temporary accommodation” and “enclosures where there is loud or excessive noise”. There were no instances where all the zoos were marked as “unacceptable” for a question in this section. However, “quarantine implementation on arrival” was rated as “unacceptable” or “questionable” at all the facilities.

#### 3.1.3. Animal Health

Animal health was one of the largest sections of the survey, and for the majority of the questions, the modal score was 3 (acceptable) (Box S3). This included the “response time between animal health problems noticed and veterinary treatment”, “secure management of vet medicines”, “record keeping of keeper observations” and “appropriate disposal of deceased animals”. There were no questions where all the zoos scored 1 (unacceptable); however, there were a number of questions (e.g., “quarantine facilities”, “preventative medicine programme”, “pest control programme” and “review of clinical records”) where a minimum of 1 (unacceptable) was scored. Modal scores of 1 (unacceptable) were generally given for questions which related to ethics and protocols for euthanasia. All the surveyed institutions were under the supervision of a veterinarian, and where recorded, “communication between the vet and the animal care team” was scored as “acceptable”.

#### 3.1.4. Animal Behaviour

There were no questions where all the assessed zoos scored 3 (acceptable) or 1 (unacceptable) (Box S4). Areas where a modal score of 3 (acceptable) were recorded included “animals being handled by trained personnel” and the “provision of separate accommodation for pregnant mothers or those with young”. Unacceptable areas (modal score of 1) related to “lone housing of some social species” and “the use of animals in performances”. Some areas of concern (the presence of a score of 1 for at least one collection) that may impact animal welfare included the “physical punishment of animals”, “management practices which don’t prevent undue dominance” and “unregulated feeding of animals by the public”.

#### 3.1.5. Perceived Mental State of the Animals

There were no questions where all the assessed zoos scored 3 (acceptable) or 1 (unacceptable) (Box S5). The “observation of positive animal behaviors” was good; however, 10 of the 11 zoos that could be assessed against this criterion scored 2 (questionable). The modal value of 3 (acceptable) for the “tethering of animals” denotes that no animals were tethered or restrained (other than being in a cage/enclosure); however, there are a number of areas which could be improved in this category, including the “provision of enrichment”.

#### 3.1.6. Animal Record Keeping

Areas of good practice in relation to animal records included up-to-date records being held for all animals and legal and ethical disposition activities (Box S6). However, two facilities scored 1 (unacceptable) on at least 50% of the questions that were answered in this section. All but one facility demonstrated legal and ethical acquisition and disposition activities.

#### 3.1.7. Staff Health and Safety

The modal score across all five of the health and safety-related questions was 1 (unacceptable) (Box S7). Areas of particular concern were the “lack of procedures relating to dangerous animal escape” and “lack of practice of emergency protocols”. For both of these questions, 5/11 of the surveyed zoos scored “unacceptable”.

#### 3.1.8. Personnel Working at the Zoo

Personnel were considered to be adequately directed, but in some instances, staff were not considered to be up to date with developments in their field, nor was there the provision of staff training and further development (minimum scores of 1 (unacceptable) were recorded in relation to these questions) (Box S8).

#### 3.1.9. Other

The modal score across all three questions in this section was 3 (acceptable) (Box S9). “Facilities are supported financially” and “storage and disposal of animal waste” were appropriate. All the zoos were fully compliant for the storage and disposal of animal waste.

### 3.2. Principal Component Analysis

The PCA yielded five components with eigenvalues > 1 (Table 1), which accounted for 75% of the total variance. Bartlett’s Test of Sphericity gave *p* < 0.001. The loadings of the questions onto each component are presented in Table 1. Cronbach’s alpha revealed good internal consistency for each component.

## 4. Discussion

Zoological facilities range widely in scope, appearance and resident populations; however, no matter their differences or locations, the animals in zoos should be afforded the opportunity to thrive. Maintaining animals in human care carries with it a burden of responsibility: we have the ethical obligation to consider the welfare of these animals and provide them with environments that enable positive welfare for all individuals. Overall (Figure 1), the lowest-scoring topics (high numbers of “unacceptable” or “questionable” and low numbers of “acceptable” scores) across the zoos were animal behaviour, animal mental state and staff health and safety. From investigating these further, it seems that there are three main aspects to consider: advancing primary care for animals, the available scientific literature and cultural barriers.

### 4.1. Aspects for Consideration

#### 4.1.1. Advancing Primary Care

Many concerns relate to the advancement of appropriate direct and indirect primary care. Primary care is considered to be the activities that underpin the provision of the physical needs of an animal and are encapsulated within operational management duties [37], such as provision for appropriate animal behaviour and, further on from this, the provision for positive mental states within the animals housed. Scientific advances on captive animal behaviour and the provision of positive experiences for zoo animals are increasing [38]. However, it may be that this knowledge is not accessible in developing countries or possibly not fully understood. For example, due to language barriers or access to resources, the Five Domains model [16] may not have been taught or available in these developing countries. However, with the newer version [17] now available with open access, this may enable easier accessibility. In addition, these advances in scientific animal care and management may not yet have been incorporated into operational management protocols and procedures at these zoos, or by the relevant national or international regulations, zoo and aquarium association guidelines or internal constitutions or bylaws. These standard operating procedures inform the “duty of care”, including monitoring and evaluation, that is required to ensure good animal husbandry and welfare. Without effective regulations in place, whether as a result of a lack of legal oversight, misguidance or simply a lack of consideration of animal protection, there is often no consistent supervision for zoos and aquariums to instruct them regarding these procedures and direct the duty of care required.

We would recommend that all staff working with animals in any zoo have access to training in the most recently recognized best-practice standards in primary animal care. This could be through links with accredited regional, national or international associations, but it is important to realize that the zoos in more need of this knowledge may not be able to afford membership of these associations or have the ability to effectively network with or within them and take full advantage of their guidance. Alternatively, it may be that zoo associations are able to set up partnerships between member and non-member zoos as a means of improving primary care provision considering the cultural needs of those locations involved, a programme that Wild Welfare now includes as part of their support. In addition, in many developing countries, legislation may not cover animal welfare in zoos, if they have animal welfare legislation in place at all [39]. With supportive legislation in place, it might become more pressing to ensure that animals in zoos are receiving the primary care that is necessary to improve welfare conditions.

#### 4.1.2. Available Scientific Literature

There is a paucity of literature on animal care and welfare available to the facilities and staff. An animal’s quality of life is driven by the owner’s/keeper’s understanding of the needs of the animals [33]. In many of these captive facilities, there is a lack of a basic understanding of species biology, behaviour and what constitutes animal welfare, and an inability to research or gather information. While there are a number of publicly available species-specific guidelines on animal welfare standards, for example, the Association of Zoos and Aquariums Care Manuals [40], these should be considered of limited use due to language barriers, online access, or the cultural implications of practice. Many evidence-based animal welfare measures and recommendations from the past decade remain inaccessible, with subscription or download fees a requirement that the majority of zoos cannot afford. Furthermore, despite the welfare of animals under human care being a moral issue, it is governed by scientific values [41] that might not be understood or accessible to the relevant people.

We would recommend that zoo welfare researchers focus research efforts on evidence-based management (research that provides recommendations as to how the animal’s welfare can be maintained or improved in order to share best practice between institutions) and are encouraged or incentivized to publish in open access journals such as PLOS ONE [42], Animals [43], the Journal of Zoo and Aquarium Research [44] or Lemur News [45], for example. For this to be financially viable due to some open access journals being subject to publication fees, it might be feasible to collaborate with universities, where funding and expertise in the area of research may be available (see review [46]). In addition, some universities have “gold membership” to certain publication libraries, thereby waiving the open access fee and allowing published literature to be open access to all, including those who do not have subscriptions.

#### 4.1.3. Cultural Barriers

Behavioural and learning barriers are present in developing country zoos, which may inhibit change or the application of practices that improve animal welfare, either through direct prevention or as a by-product of insufficient knowledge. The staff members who care for animals are important elements of animals’ environments, and the quality of the relationships with the humans who care for them has an impact on the welfare of the animals [2,47]. While some institutions provide expert welfare assistance to raise the standards of animal care, others are less enthusiastic [48]; it is often these facilities that are most in need of support. This institutional culture and attitude limits learning opportunities, even for individuals who have shown a willingness or desire to make improvements in animal care practice. This has the potential to reduce the accessibility of such values, limiting engagement on a level that incorporates learning opportunities.

An animal’s welfare is directly dependent upon the quality of life they experience, and sub-optimal husbandry conditions can result in poor mental and physical health [33]. The implementation of good management techniques, including the provision of varied environmental enrichment and standards that not only meet needs and requirements but also promote positive physical and psychological health, is fundamental for the care of wild animals in zoos. This “duty of care” requires that captive facilities take steps so that animal-management practices address specific animal needs and impact areas that promote positive experiences and prevent or minimize unnecessary negative experiences [49]. Zoos must provide environments not only in which animals can cope, but in which animals can thrive [50]. A complex understanding of species-specific behaviour and required care is required by animal care staff for positive welfare to be achieved, and that requires engagement in more detailed literature. This engagement only comes about with an interest in the species and a desire to improve conditions for individual animals.

Identifying the barriers to developing positive relationships between the animals and their care givers is a critical element of improving animal welfare standards. Promoting long-term changes in animal care requires a change in the attitudes, behaviour and knowledge within the institution’s culture. It is imperative that a simple, user friendly and facility-driven primary care assessment framework is used, with or without external support from a partner or association. Ownership and leadership in such a framework can stimulate a more engaged approach amongst all staff to animal care and welfare.

We recognize that most relevant journals, including those focused on general zoo research or welfare science, are published in English. We would recommend that the relevant zoo associations and interested parties (local universities, for example) collaborate to interpret the scientific outputs (e.g., the most relevant findings for zoos over the course of a year) to ensure they are understandable to the lay person, translated into the local language and distributed to zoos so that they are provided with up-to-date information and are able to make animal management decisions based on appropriate, up-to-date scientific knowledge.

### 4.2. Audit Evaluation

The current Wild Welfare Audit comprises 110 questions separated into nine topic areas; however, due to this audit being originally designed as a tool for comparing facilities to the WW core fundamental standards [34], there are some repetitions in the information gathered by the questions, for example, “are the animals generally in good body condition?” and “are there animals that are underweight?”, both from the nutrition topic, and “do the animals appear to be in good health?” from the animal health topic. The components identified through the PCA may provide more appropriate groupings of questions, to enable a more streamlined and efficient approach to future surveying (Table 2).

Our analysis of the WW Audit showed that, whilst it does not prioritize the promotion of positive experiences as its assessment criteria, it consistently questions a wide range of management practices pertaining to primary care that can have a direct and indirect impact on an animal’s welfare [23] and demonstrates clear common welfare concerns across the surveyed facilities. It was noted that there were a higher number of questions within the animal health topic area. Any bias towards questions pertaining to animal health may also be due to the pre-established “snap-shot” audit approach that relies on a range of physical health indicators in domestic species [51], including body condition scores, signs of disease or lameness [52], and the ease of assessment with more quantifiable data. Good basic health care, in terms of the five basic needs being met, is usually taken for granted within leading zoos and aquariums, and thus, more focus can be given to behavioural outcomes and developing understanding of animals’ affective states, but a lack of expertise means that within non-affiliate facilities, questioning animal health care activities must be a serious consideration.

Although species-specific welfare assessments can be beneficial in assessing the behavioural and physical needs of a species, there is scope for the use of more broad, generic approaches suitable for zoos, such as the Wild Welfare Audit or others developed for more advanced and well-supported zoos [31]. The use of generic welfare assessments can avoid the common taxon bias seen in many areas of zoo science [38,53,54]. This whole-collection approach can also address general management practices and current and future planning, which can have an impact on a large number of individual animals, but is not always considered under species-specific welfare measures. Furthermore, they enable the prioritization of management for welfare needs across all taxa, not just the most high-profile or well-studied in the collection [55]. A systematic assessment being undertaken by impartial and knowledgeable auditors can generate dialogue and engagement through an appropriate, evidenced-based approach, regarding the necessity for collection planning, that prioritizes animal welfare, while maintaining the fundamental and/or cultural values of that particular facility. After WW training, zoo staff are encouraged to conduct their own welfare audits. It is important that zoo staff are engaged with this process, but we also need to be mindful of the possibility of biased results, either positively or negatively [56].

This research has helped to show that although there are zoos that may score high on zoo welfare audits and have access to the support and knowledge of regional, national and global zoo associations as well as up-to-date published literature that is accessible and understandable, there are many zoos that do not. Evidence-based zoo animal husbandry and welfare has developed considerably in the last 10 years [38], but there is a clear need for bridging the gap and sharing knowledge of simple and applicable positive welfare techniques used in more advanced zoos with developing country zoos that want or need to improve their facilities and husbandry standards.

## 5. Conclusions

The aim of this research was to investigate if there were common welfare concerns associated with zoo animal provision across a range of zoos in developing countries. The results revealed that there were issues linked to animal behaviour and an animal’s mental state as well as human health and safety provisions. When investigated further, these concerns were found to be associated with the lack of advancing primary animal care, which may be due to historical and cultural differences in husbandry routines as well as a lack of knowledge and availability of both practical learning opportunities and up-to-date publications on zoo welfare science. It is important that knowledge transfer on topics such as zoo welfare science is supported and encouraged, to ensure that all zoos, irrespective of their culture or location, are able to improve standards of animal care and welfare through evidence-based management, to ensure animal welfare is optimized.

## Figures and Tables

**Table 1:** Results of principal component analysis from the Wild Welfare Audit.

Question	Component				
	1 α = 0.959	2 α = 0.839	3 α = 0.651	4 α = 0.815	5 α = 0.836
Are the animals generally in good body condition?	0.895				
Are there any animals that are underweight?		0.811			
Are there any animals that are overweight?	0.634				
Do all animals have ready access to plenty of clean, potable water?			0.919		
Is the quantity of food provided for the animals documented, adequate and the consumption thereof, monitored?			0.647		
Does the provided food meet the specific nutritional requirements of each species and of each individual?	0.803				
Is dietary supplementation given?	0.726				
Are supplies of food and drink prepared under hygienic conditions?		0.968			
Is food stored correctly to protect it from damp, deterioration and contamination by pests?		0.968			
Are perishable foods kept refrigerated?		0.941			
Are there enough food and drinking sites so as to be accessible to every animal within a particular enclosure?					0.652
Are food and drink provided in such a way that they meet the biological and behavioral needs of the animal?					0.644
Are feeding enrichment techniques used?	0.722				
Are the diets of the animals reviewed regularly?	0.711				
Are there feeding protocols in place should hand rearing be necessary?					
Do the majority of the enclosures appear to be clean and well maintained?			0.842		
Are the enclosures free from vegetation or other items that would aid animal escape?		0.831			
Is the drainage of the majority of enclosures safe, efficient and appropriate?	0.664				
Can personnel service all enclosures in a manner that is both safe to themselves and the inhabitants?			0.677		
Do the majority of enclosure environments provide for the well-being of the animals throughout the year?	−0.681				
Are the majority of the enclosure substrates, design features and furniture sufficient to provide enough shelter and refuge for all specimens displayed, including those kept in multi-species exhibits?					0.722
Are routine veterinary examinations performed prior to transport?			0.665		
Is quarantine implemented on arrival of acquisitions?					
Is the response time between noticing/reporting an animal health problem and the receipt of appropriate veterinary care adequate?		0.947			
Are the biosecurity measures in place sufficient and suitable?				0.727	
Do the animals appear to be in good health, with no obvious signs or injury or illness?	0.743				
Do mutilation procedures appear to have been carried out on any of the animals?					
Is the frequency of visual inspection of the animals by keeper staff suitable and the protocol for reporting health concerns effective?	0.715				
Are keeper observations of general animal health and behavior recorded?	0.843				
Is the frequency of routine clinical examinations for all of the animals appropriate?	0.793				
Is there a suitable preventative medicine programme in place?	0.800				
Does the facility normally perform necropsies?		0.653			
Are suitable samples from necropsies submitted for pathological analysis?		0.657			
Is there a safe and effective programme for the control of pests and where necessary, predators?	0.832				
Does management practice ensure that an uncontrolled build-up of parasites and other pathogens is prevented?				0.695	
Does the facility maintain up-to-date veterinary records on the health of individual animals within the collection?		0.781			
Does a review of clinical records, animal health management and disease issues take place?					0.625
Is euthanasia carried out under veterinary supervision, or by a competent, senior staff member properly trained who has access to the necessary equipment and facilities?				0.870	
Where appropriate, are the animals maintained in social groups of suitable composition (e.g., number, age and sex ratio)?				0.724	
Are there any naturally social species currently housed in enclosures on their own?	0.808				
Is separate accommodation provided where appropriate for pregnant mothers and animals with young?			0.814		
Are the majority of the enclosure substrates, design features and furniture sufficient to provide for the behavioral needs of all individuals displayed, including those kept in multi-species exhibits?		−0.569			
Is the regulated feeding of specific animals by visitors permitted?		0.688			
Does the facility have animal demonstrations, shows and/or animal rides or undertake any form of animal contact?			−0.829		
Are animals handled only by or under the supervision of authorized personnel?	0.675				
Are the animals’ welfare needs appropriately managed with due regard to the requirements of the viewing public?					
Is environmental and behavioral enrichment regularly provided?	0.815				
Are the animals generally bright, alert and interested and engaged in their surroundings?	0.855				
Are any of the animals restrained or tethered at any time?			0.652		
Are up to date records (including husbandry details, daily behavioral observations, etc) held for all individual animals?	0.915				
Is the system of recording information easy to search, secure and fit for purpose?	0.915				
Are there records kept of the movement of animals into and out of the institution?	0.683				
Can all of the animals held at the institution be individually identified?			0.609		
Is animal population management regularly reviewed?	0.648				
Are there procedures and equipment in place in the event of a dangerous animal escape?	0.830				
Are the emergency protocols practiced and if so, how often?	0.830				
Are records kept in the event of an animal escape/attack?	0.899				
Do staff receive training in animal health, disinfection principles and hygiene practices?	0.613				
Does the facility have clear procedures for working with hazardous animals?	0.821				
Does the facility have continuing financial support?			0.827		
Does the total financial support appear to be adequate to meet the needs of the facility?	0.764				
Is the staffing level appropriate to provide the required standards of animal husbandry and care?		0.973			
Do staff members regularly meet to discuss problems and possible solutions?	0.698				
In general, do there appear to be good working relations in the zoos?		0.966			
Are animal care staff up to date with developments in their field of expertise?	0.825				
Is there provision for staff training and further development?	0.683				

**Table 2:** Summary of recommended questions to be included in future assessments of zoo animal welfare in developing country zoos.

PCA Grouping	Current Topic	Relevant Question Themes to Be Included
1	Nutrition	Body condition scores
		Food/drink meets biological and nutritional needs
		Food/drink accessible by all
	Animal health	Health checks at appropriate intervals
		Preventative medicines programme
		Pest/predator control programme
		Dietary review
	Environment	Appropriate drainage
	Behavior	Appropriate social groups
	Mental health	Enriched/engaging environments
	Records	Are records up to date, readily accessible and covering all animal moves?
		Is animal management reviewed?
	Health and safety	Are dangerous animal procedures in place?
		Staff training in health/hygiene
	Personnel	Appropriate staff training and development opportunities
2	Nutrition	Appropriate storage and preparation of foodstuffs
	Animal health	Appropriate veterinary care
		Veterinary records up to date
		Necropsies/analysis performed
	Behavior	Behavioral needs catered for
		Regulated visitor feeding
	Personnel	Appropriate staffing levels
3	Nutrition	Appropriate, monitored, feed and water provision
	Environment	Clean/well maintained enclosures
		Enclosures safely accessible
		Routine examinations prior to transport
	Behavior	Separate accommodation where required for pregnant mothers/nursing animals
		Animal demonstrations/animal rides/animal contact
	Mental health	Tethering/restraint of animals
	Records	Identification of animals possible
	Other	Financial support
4	Animal health	Biosecurity measures which prevent parasite/pathogen build up
		Euthanasia carried out under veterinary supervision, or by competent trained staff
5	Nutrition	Food/drink provision meets behavioral and physical needs of all of the animals
	Environment	Appropriate shelter and refuge in enclosures
	Animal health	Reviews of clinical records, animal health management and disease issues

## Supplementary Materials

The following are available online at https://www.mdpi.com/2076-2615/10/11/2101/s1. Supplementary Materials File 1: Box S1: A breakdown of scores given in the Wild Welfare Audit on questions related to nutritional provision. Box S2. A breakdown of scores given in the Wild Welfare Audit on questions related to the environment in which animals are housed. Box S3. A breakdown of scores given in the Wild Welfare Audit on questions related to animal health. Box S4. A breakdown of scores given in the Wild Welfare Audit on questions related to animal behaviour. Box S5. A breakdown of scores given in the Wild Welfare Audit on questions related to the perceived mental state of the animals. Box S6. A breakdown of scores given in the Wild Welfare Audit on questions related to the standard of animal record keeping. Box S7. A breakdown of scores given in the Wild Welfare Audit on questions related to staff health and safety. Box S8. A breakdown of scores given in the Wild Welfare Audit on questions on information relating to personnel working at the zoo. Box S9. A breakdown of scores given in the Wild Welfare Audit on questions related to the other questions in the audit. Supplementary Materials File 2: Wild Welfare Audit Used for Assessing Zoo Welfare.
