# Supplementary material for: Using Zoo Welfare Assessments to Identify Common Issues in Developing Country Zoos
Source: Animals (Basel). 2020 Nov 12;10(11):2101. doi: 10.3390/ani10112101 (PMC7696472; doi:10.3390/ani10112101)
Supplement: Supplementary file 1 [file animals-10-02101-s001.pdf]

Supplementary Materials File 1

Nutrition

Areas of concern across zoos [modal value 1]:

- Are feeding enrichment techniques used?
- Are live vertebrate animals offered as food to any animal?

Areas of questionable practice [modal value 2]:

- Is dietary supplementation given?
- Are food and drink provided in such a way that they meet the biological and behavioural needs of the animal?
- Are there feeding protocols in place should hand rearing be necessary?

Areas of good practice across zoos [modal value 3]:

- Are the animals generally in good body condition?
- Are there any animals that are underweight?
- Are there any animals that are overweight?
- Do all animals have ready access to plenty of clean, potable water?
- Is food sourced from a reputable supplier, ensuring that it is free from any contaminants?
- Is the quality of animal foodstuffs adequate and acceptable?
- Is the quantity of food provided for the animals documented, adequate and the consumption thereof, monitored?.
- Does the provided food meet the specific nutritional requirements of each species and of each individual?
- Are supplies of food and drink prepared under hygienic conditions?
- Is food stored correctly to protect it from damp, deterioration and contamination by pests?
- Are perishable foods kept refrigerated?
- Is the manner of feeding safe for both the animals and the staff?
- Are there enough food and drinking sites so as to be accessible to every animal within a particular enclosure?
- Are the diets of the animals reviewed regularly?

Areas of concern across zoos [at least one zoo is scoring 1 for this measure]

- Are there any animals that are underweight?
- Are there any animals that are overweight?
- Do all animals have ready access to plenty of clean, potable water?
- Is the quantity of food provided for the animals documented, adequate and the consumption thereof, monitored?
- Are feeding enrichment techniques used?
- Are the diets of the animals reviewed regularly?
- Are there feeding protocols in place should hand rearing be necessary?
- Are live vertebrate animals offered as food to any animal?

Areas which are considered fully unacceptable [all zoos scored 1]

- Are live vertebrate animals offered as food to any animal?

Areas which are considered fully acceptable [all zoos scored 3]

- Is food sourced from a reputable supplier, ensuring that it is free from any contaminants?
- Is the quality of animal foodstuffs adequate and acceptable?
- Is the manner of feeding safe for both the animals and the staff?

Box S1. A breakdown of scores given on the wild welfare audit on questions related to nutritional provision.

Environment

Areas of concern across zoos [modal value 1]:

- Are animals kept in temporary accommodation? If yes, is their situation regularly and appropriately assessed?

Areas of questionable practice [modal value 2]:

- Does management practice ensure that physical carrying capacity is not overburdened?
- Is the drainage of the majority of enclosures safe, efficient and appropriate?
- In aquatic enclosures are appropriate water quality parameters measured and controlled?
- Do the majority of enclosure environments provide for the well-being of the animals throughout the year?
- Is there adequate ventilation and appropriate lighting in indoor areas and holding areas?
- Are the majority of the enclosure substrates, design features and furniture sufficient to provide enough shelter and refuge for all specimens displayed, including those kept in multi-species exhibits?
- Is quarantine implemented on arrival of acquisitions?

Areas of good practice across zoos [modal value 3]:

- Do the majority of the enclosures appear to be clean and well maintained?
- Is the total number of animals accommodated appropriate for the area of land occupied by the institution and the available resources?
- Are the enclosures free from vegetation or other items that would aid animal escape?
- Are gates/doors to enclosures containing hazardous animals securely locked at all times?
- Do gates/doors to enclosures appear to be as strong and effective in containing the animals as the rest of the enclosure barrier?
- Can personnel service all enclosures in a manner that is both safe to themselves and the inhabitants?
- Are the environmental temperature and humidity levels maintained appropriately for the animals?
- Are any of the enclosures located where there is loud or excessive noise?
- Are there facilities for crating and transporting animals?
- Are routine veterinary examinations performed prior to transport?

Areas of concern across zoos [at least one zoo is scoring 1 for this measure]

- Does management practice ensure that physical carrying capacity is not overburdened?
- Is the drainage of the majority of enclosures safe, efficient and appropriate?
- Can personnel service all enclosures in a manner that is both safe to themselves and the inhabitants?
- Are animals kept in temporary accommodation? If yes, is their situation regularly and appropriately assessed?
- Do the majority of enclosure environments provide for the well-being of the animals throughout the year?
- Is there adequate ventilation and appropriate lighting in indoor areas and holding areas?
- Are any of the enclosures located where there is loud or excessive noise?
- Are the majority of the enclosure substrates, design features and furniture sufficient to provide enough shelter and refuge for all specimens displayed, including those kept in multi-species exhibits?
- Is quarantine implemented on arrival of acquisitions?

Areas which are considered fully unacceptable [all zoos scored 1]

- None

Areas which are considered fully acceptable [all zoos scored 3]

- Do gates/doors to enclosures appear to be as strong and effective in containing the animals as the rest of the enclosure barrier?
- Are there facilities for crating and transporting animals?

Box S2. A breakdown of scores given on the wild welfare audit on questions related to the environment in which animals are housed.

Health

Areas of concern across zoos [modal value 1]:

- Are the quarantine facilities and quarantine protocols appropriate?
- Are there written protocols for the euthanasia of animals?
- Is euthanasia carried out under veterinary supervision, or by a competent, senior staff member properly trained and experienced in the techniques used, who has access to the necessary equipment and facilities and who is available at all times?
- Is there any form of ethical review or oversight?

Areas of questionable practice [modal value 2]:

- Are the biosecurity measures in place sufficient and suitable?
- Do the animals appear to be in good health, with no obvious signs or injury or illness?
- Is there a safe and effective programme for the control of pests and where necessary, predators?
- Does management practice ensure that an uncontrolled build-up of parasites and other pathogens is prevented?
- Are the circumstances mandating euthanasia or humane killing appropriate?

Areas of good practice across zoos [modal value 3]:

- Is the animal collection under the supervision of a veterinarian?
- Does the level of veterinary supervision and care provided appear to be adequate for the size of the institution and number of animals accommodated?
- Is there clear effective communication between the veterinarian and the institution's animal care team?
- Is the response time between noticing/reporting an animal health problem and the receipt of appropriate veterinary care adequate?
- Is the veterinary examination/treatment room adequate and does it have suitable facilities to meet the needs of the collection?
- Is there proper, secure management of all veterinary medicines?
- Do mutilation procedures appear to have been carried out on any of the animals?
- Is the frequency of visual inspection of the animals by keeper staff suitable and the protocol for reporting health concerns effective?
- Are keeper observations of general animal health and behaviour recorded?
- Is the frequency of routine clinical examinations for all of the animals appropriate?
- Is there a suitable preventative medicine programme in place?
- Does the facility normally perform necropsies?
- Are suitable samples from necropsies submitted for pathological analysis?
- Is the area where necropsies are performed suitable?
- Are deceased animals stored away from food and disposed of appropriately?
- Is there well maintained and appropriate animal capture equipment available on site, along with a sufficient number of trained staff to use it?
- Does the facility maintain up-to-date veterinary records on the health of individual animals within the collection?
- Does a review of clinical records, animal health management and disease issues take place?

Health cont'd

Areas of concern across zoos [at least one zoo is scoring 1 for this measure]

- Does the level of veterinary supervision and care provided appear to be adequate for the size of the institution and number of animals accommodated?
- Is the veterinary examination/treatment room adequate and does it have suitable facilities to meet the needs of the collection?
- Are the quarantine facilities and quarantine protocols appropriate?
- Are the biosecurity measures in place sufficient and suitable?
- Are keeper observations of general animal health and behaviour recorded?
- Is the frequency of routine clinical examinations for all of the animals appropriate?
- Is there a suitable preventative medicine programme in place?
- Is there a safe and effective programme for the control of pests and where necessary, predators?
- Does management practice ensure that an uncontrolled build-up of parasites and other pathogens is prevented?
- Is there well maintained and appropriate animal capture equipment available on site, along with a sufficient number of trained staff to use it?
- Does the facility maintain up-to-date veterinary records on the health of individual animals within the collection?
- Does a review of clinical records, animal health management and disease issues take place?
- Are there written protocols for the euthanasia of animals?
- Is euthanasia carried out under veterinary supervision, or by a competent, senior staff member properly trained and experienced in the techniques used, who has access to the necessary equipment and facilities and who is available at all times?
- Are the circumstances mandating euthanasia or humane killing appropriate?
- Is there any form of ethical review or oversight?

Areas which are considered fully unacceptable [all zoos scored 1]

- None

Areas which are considered fully acceptable [all zoos scored 3]

- Is the animal collection under the supervision of a veterinarian?
- Is there clear effective communication between the veterinarian and the institution's animal care team?
- Are deceased animals stored away from food and disposed of appropriately?

Box S3. A breakdown of scores given on the wild welfare audit on questions related to animal health.

Behaviour

Areas of concern across zoos [modal value 1]:

- Are there any naturally social species currently housed in enclosures on their own?
- Does the facility have animal demonstrations, shows and/or animal rides or undertake any form of animal contact?

Areas of questionable practice [modal value 2]:

- Where appropriate, are the animals maintained in social groups of suitable composition (eg number, age and sex ratio)?
- Does management practice ensure that persistent and unresolved conflict is avoided?
- Are the majority of the enclosure substrates, design features and furniture sufficient to provide for the behavioural needs of all individuals displayed, including those kept in multi-species exhibits?
- If there are free ranging animals on site (eg peacocks, guinea fowl), are they monitored and is there a documented husbandry and management protocol for their care?
- Do indoor, outdoor and holding enclosure areas all allow for normal behaviour patterns and ranges of movements to be expressed?
- Does the handling of animals by personnel appear to be consistent with the animal's welfare?
- If there are any animal-training programmes in place at the institution, are they documented and appropriate in technique, duration and purpose?
- Is physical punishment of the animals ever used?
- Is there regular monitoring and review of animal contact and training programmes?

Areas of good practice across zoos [modal value 3]:

- Does management practice ensure that undue dominance by individuals is avoided?
- Is separate accommodation provided where appropriate for pregnant mothers and animals with young?
- Does the unregulated feeding of the animals by visitors take place?
- Is the regulated feeding of specific animals by visitors permitted? If so, under what specific circumstances is it allowed and how is it supervised, controlled and managed?
- Are animals handled only by or under the supervision of authorised personnel?

Areas of concern across zoos [at least one zoo is scoring 1 for this measure]

- Are there any naturally social species currently housed in enclosures on their own?
- Does management practice ensure that undue dominance by individuals is avoided?
- Does management practice ensure that persistent and unresolved conflict is avoided?
- Are the majority of the enclosure substrates, design features and furniture sufficient to provide for the behavioural needs of all individuals displayed, including those kept in multi-species exhibits?
- Do indoor, outdoor and holding enclosure areas all allow for normal behaviour patterns and ranges of movements to be expressed?
- Does the unregulated feeding of the animals by visitors take place?
- Is the regulated feeding of specific animals by visitors permitted? If so, under what specific circumstances is it allowed and how is it supervised, controlled and managed?
- Does the facility have animal demonstrations, shows and/or animal rides or undertake any form of animal contact?
- Does the handling of animals by personnel appear to be consistent with the animal's welfare?
- Is physical punishment of the animals ever used?
- Is there regular monitoring and review of animal contact and training programmes?

Areas which are considered fully unacceptable [all zoos scored 1]

- None

Areas which are considered fully acceptable [all zoos scored 3]

- None

Box S4. A breakdown of scores given on the wild welfare audit on questions related to animal behavior.

Mental health

Areas of concern across zoos [modal value 1]:

- Is environmental and behavioral enrichment regularly provided?
- Are negative animal behaviours (eg over-grooming, avoidance, stereotyping, hyper-aggression, apathy, etc) observed?

Areas of questionable practice [modal value 2]:

- Are the animal's welfare needs appropriately managed with due regard to the requirements of the viewing public?
- Are the animals generally bright, alert and interested and engaged in their surroundings?
- Are positive animal behaviours (eg play, exploration, rest, normal feeding, etc) observed?

Areas of good practice across zoos [modal value 3]:

- Are any of the animals restrained or tethered at any time?

Areas of concern across zoos [at least one zoo is scoring 1 for this measure]

- Are the animal's welfare needs appropriately managed with due regard to the requirements of the viewing public?
- Is environmental and behavioral enrichment regularly provided?
- Are the animals generally bright, alert and interested and engaged in their surroundings?
- Are negative animal behaviours (eg over-grooming, avoidance, stereotyping, hyper-aggression, apathy, etc) observed?
- Are any of the animals restrained or tethered at any time?

Areas which are considered fully unacceptable [all zoos scored 1]

- None

Areas which are considered fully acceptable [all zoos scored 3]

- None

Box S5. A breakdown of scores given on the wild welfare audit on questions related to the perceived mental state of the animals.

Animal records

Areas of concern across zoos [modal value 1]:

- Does the facility have any form of collection plan?

Areas of questionable practice [modal value 2]:

- Can all of the animals held at the institution be individually identified? If so, what methods of individual animal identification are used?
- Is animal population management regularly reviewed?

Areas of good practice across zoos [modal value 3]:

- Are up to date records (including husbandry details, daily behavioural observations, etc) held for all individual animals?
- Is the system of recording information easy to search, secure and fit for purpose?
- Are there records kept of the movement of animals into and out of the institution?
- Are acquisition and disposition activities legal and ethical?

Areas of concern across zoos [at least one zoo is scoring 1 for this measure]

- Are up to date records (including husbandry details, daily behavioural observations, etc) held for all individual animals?
- Is the system of recording information easy to search, secure and fit for purpose?
- Are there records kept of the movement of animals into and out of the institution?
- Does the facility have any form of collection plan?
- Can all of the animals held at the institution be individually identified? If so, what methods of individual animal identification are used?
- Is animal population management regularly reviewed?

Areas which are considered fully unacceptable [all zoos scored 1]

- None

Areas which are considered fully acceptable [all zoos scored 3]

- None

Box S6. A breakdown of scores given on the wild welfare audit on questions related to the standard of animal record keeping.

Health & safety

Areas of concern across zoos [modal value 1]:

- Are there procedures and equipment in place in the event of a dangerous animal escape?
- Are the emergency protocols practised and if so, how often?
- Are records kept in the event of an animal escape/attack?
- Do staff receive training in animal health, disinfection principles and hygiene practices?
- Does the facility have clear procedures for working with hazardous animals?

Areas of questionable practice [modal value 2]:

- None

Areas of good practice across zoos [modal value 3]:

- None

Areas of concern across zoos [at least one zoo is scoring 1 for this measure]

- Are there procedures and equipment in place in the event of a dangerous animal escape?
- Are the emergency protocols practised and if so, how often?
- Are records kept in the event of an animal escape/attack?
- Do staff receive training in animal health, disinfection principles and hygiene practices?
- Does the facility have clear procedures for working with hazardous animals?

Areas which are considered fully unacceptable [all zoos scored 1]

- None

Areas which are considered fully acceptable [all zoos scored 3]

- Is the staff adequately directed?

Box S7. A breakdown of scores given on the wild welfare audit on questions related to staff health and safety.

Personnel

Areas of concern across zoos [modal value 1]:

- None

Areas of questionable practice [modal value 2]:

- Are animal care staff up to date with developments in their field of expertise?

Areas of good practice across zoos [modal value 3]:

- Is the staff adequately directed?
- Is the staffing level appropriate to provide the required standards of animal husbandry and care?
- Do staff members regularly meet to discuss problems and possible solutions?
- In general, do there appear to be good working relations in the zoos?
- Is there provision for staff training and further development?

Areas of concern across zoos [at least one zoo is scoring 1 for this measure]

- Are animal care staff up to date with developments in their field of expertise?
- Is there provision for staff training and further development?

Areas which are considered fully unacceptable [all zoos scored 1]

- None

Areas which are considered fully acceptable [all zoos scored 3]

- Is the staff adequately directed?

Box S8. A breakdown of scores given on the wild welfare audit on questions related to 'other'.

Physical facilities

Areas of concern across zoos [modal value 1]:

- None

Areas of questionable practice [modal value 2]:

- None

Areas of good practice across zoos [modal value 3]:

- Are there adequate provisions for the proper storage and disposal of animal waste?
- Does the facility have continuing financial support?
- Does the total financial support appear to be adequate to meet the needs of the facility?

Areas of concern across zoos [at least one zoo is scoring 1 for this measure]

- None

Areas which are considered fully unacceptable [all zoos scored 1]

- None

Areas which are considered fully acceptable [all zoos scored 3]

- None

Box S9. A breakdown of scores given on the wild welfare audit on questions on information relating to personnel working at the zoo.

Supplementary Materials File 2

LIVE COLLECTION PRELIMINARY ASSESSMENT FORM

Caveat:

Management practices Wild Welfare considers unacceptable and which Wild Welfare recommends should cease in order to provide for good animal welfare include:

- The feeding of live vertebrate animals to any animal
- Training techniques involving physical punishment, or training practices that compromise the animal's physical or behavioural health, development or psychological well-being
- Animal demonstrations that are detrimental to the physical or psychological well-being of the animals
- Confinement in barren enclosures that severely restrict physical movement and compromise psychological wellbeing
- Mutilation procedures for cosmetic purposes or to make an animal safe for handling
- Unregulated feeding of the animals by visitors

Captive wild animal management must aim to provide suitable environments that provide for good welfare for all captive species; it is unacceptable to maintain any species in captivity in conditions that compromise their health and welfare.

Key:

A = Acceptable (requires no immediate attention)

Q = Questionable (should be improved or changed as soon as possible, but is not a critical matter)

UA = Unacceptable (must be corrected).

Nap = Not applicable

Nass= Not assessed

Rate according to the 5 criteria and give notes, explanations and expanded points for all rankings

	Veterinary Care	A	Q	UA	Nap	Nass
1	Is the animal collection under the supervision of a veterinarian?					
2	Does the level of veterinary supervision and care provided appear to be adequate for the size of the institution and number of animals accommodated?					
3	Is there clear effective communication between the veterinarian and the institution's animal care team?					
4	Is the response time between noticing/reporting an animal health problem and the receipt of appropriate veterinary care adequate?					
5	Is the veterinary examination/treatment room adequate and does it have suitable facilities to meet the needs of the collection?					
6	Is there proper, secure management of all veterinary medicines?					
7	Are the quarantine facilities and quarantine protocols appropriate?					
8	Are the biosecurity measures in place sufficient and suitable?					
9	Do the animals appear to be in good health, with no obvious signs or injury or illness?					
10	Do mutilation procedures appear to have been carried out on any of the animals?					
11	Is the frequency of visual inspection of the animals by keeper staff suitable and the protocol for reporting health concerns effective?					
12	Are keeper observations of general animal health and behaviour recorded?					
13	Is the frequency of routine clinical examinations for all of the animals appropriate?					

14	Is there a suitable preventative medicine programme in place?					
15	Does the facility normally perform necropsies?					
16	Are suitable samples from necropsies submitted for pathological analysis?					
17	Is the area where necropsies are performed suitable?					
18	Are deceased animals stored away from food and disposed of appropriately?					
19	Is there a safe and effective programme for the control of pests and where necessary, predators?					
20	Is there well maintained and appropriate animal capture equipment available on site, along with a sufficient number of trained staff to use it?					
21	Does the facility maintain up-to-date veterinary records on the health of individual animals within the collection?					
22	Does a review of clinical records, animal health management and disease issues take place?					
23	Are there written protocols for the euthanasia of animals?					
24	Is euthanasia carried out under veterinary supervision, or by a competent, senior staff member properly trained and experienced in the techniques used, who has access to the necessary equipment and facilities and who is available at all times?					
25	Are the circumstances mandating euthanasia or humane killing appropriate?					
26	Is there any form of ethical review or oversight?					
	**Animal Management**	**A**	**Q**	**UA**	**Nap**	**Nass**
27	Do the majority of the enclosures appear to be clean and well maintained?					

28	Where appropriate, are the animals maintained in social groups of suitable composition (eg number, age and sex ratio)?					
29	Are there any naturally social species currently housed in enclosures on their own?					
30	Does management practice ensure that undue dominance by individuals is avoided?					
31	Does management practice ensure that persistent and unresolved conflict is avoided?					
32	Does management practice ensure that physical carrying capacity is not overburdened?					
33	Is the total number of animals accommodated appropriate for the area of land occupied by the institution and the available resources?					
34	Does management practice ensure that an uncontrolled build-up of parasites and other pathogens is prevented?					
35	Is separate accommodation provided where appropriate for pregnant mothers and animals with young?					
36	Are animals kept in temporary accommodation? If yes, is their situation regularly and appropriately assessed?					
37	Do the majority of enclosure environments provide for the well-being of the animals throughout the year?					
38	Are the environmental temperature and humidity levels maintained appropriately for the animals?					
39	Is there adequate ventilation and appropriate lighting in indoor areas and holding areas?					
40	Are any of the enclosures located where there is loud or excessive noise?					
41	Are the majority of the enclosure substrates, design features and furniture sufficient to provide enough shelter and refuge for all					

	specimens displayed, including those kept in multi-species exhibits?					
42	Are the majority of the enclosure substrates, design features and furniture sufficient to provide for the behavioural needs of all individuals displayed, including those kept in multi-species exhibits?					
43	Is the drainage of the majority of enclosures safe, efficient and appropriate?					
44	In aquatic enclosures are appropriate water quality parameters measured and controlled?					
45	Is the equipment (including back up facilities) that is necessary to ensure correct enclosure environmental provision well maintained?					
46	Can personnel service all enclosures in a manner that is both safe to themselves and the inhabitants?					
47	If there are free ranging animals on site (eg peacocks, guinea fowl), are they monitored and is there a documented husbandry and management protocol for their care?					
	**Behaviour & Mental Health**	**A**	**Q**	**UA**	**Nap**	**Nass**
48	Do indoor, outdoor and holding enclosure areas all allow for normal behaviour patterns and ranges of movements to be expressed?					
49	Is environmental enrichment regularly provided?					
50	Are the animals generally bright, alert and interested and engaged in their surroundings?					
51	Are positive animal behaviours (eg play, exploration, rest, normal feeding, etc) observed?					
52	Are negative animal behaviours (eg over-grooming, avoidance, stereotyping, hyper-aggression, apathy, etc) observed?					
53	Are any of the animals restrained or tethered at any time?					

	**Food and food hygiene**	**A**	**Q**	**UA**	**Nap**	**Nass**
54	Are the animals generally in good body condition?					
55	Are there any animals that are underweight?					
56	Are there any animals that are overweight?					
57	Do all animals have ready access to plenty of clean, potable water?					
58	Is food sourced from a reputable supplier, ensuring that it is free from any contaminants?					
59	Is the quality of animal foodstuffs adequate and acceptable?					
60	Is the quantity of food provided for the animals documented, adequate and the consumption thereof, monitored?.					
61	Does the provided food meet the specific nutritional requirements of each species and of each individual?					
62	Is dietary supplementation given?					
63	Are supplies of food and drink prepared under hygienic conditions?					
64	Is food stored correctly to protect it from damp, deterioration and contamination by pests?					
65	Are perishable foods kept refrigerated?					
66	Is the manner of feeding safe for both the animals and the staff?					
67	Are there enough food and drinking sites so as to be accessible to every animal within a particular enclosure?					
68	Are food and drink provided in such a way that they meet the biological and behavioural needs of the animal?					
69	Are feeding enrichment techniques used?					
70	Are the diets of the animals reviewed regularly?					

71	Are there feeding protocols in place should hand rearing be necessary?					
72	Are live vertebrate animals offered as food to any animal?					
	**Animal Records**	**A**	**Q**	**UA**	**Nap**	**Nass**
73	Are up to date records (including husbandry details, daily behavioural observations, etc) held for all individual animals?					
74	Is the system of recording information easy to search, secure and fit for purpose?					
75	Does the facility have any form of collection plan?					
76	Can all of the animals held at the institution be individually identified? If so, what methods of individual animal identification are used?					
	**Enclosures & Barriers**	**A**	**Q**	**UA**	**Nap**	**Nass**
77	Are the enclosures and barriers designed, constructed and in such a condition to safely contain animals within the desired enclosures?					
78	Are the enclosures free from vegetation or other items that would aid animal escape?					
79	Are gates/doors to enclosures containing hazardous animals securely locked at all times?					
80	Do gates/doors to enclosures appear to be as strong and effective in containing the animals as the rest of the enclosure barrier?					
81	Where appropriate, are adequate standoff barriers provided to prevent direct contact between visitors and enclosures?					
	**Visitors, Animal Contact & Training**	**A**	**Q**	**UA**	**Nap**	**Nass**
82	Are the animals welfare needs appropriately managed with due regard to the requirements of the viewing public?					
83	Does the unregulated feeding of the animals by visitors take place?					

84	Is the regulated feeding of specific animals by visitors permitted? If so, under what specific circumstances is it allowed and how is it supervised, controlled and managed?					
85	Does the facility have animal demonstrations, shows and/or animal rides or undertake any form of animal contact?					
86	Are animals ever involved in animal contact situations off-site?					
87	Are animals handled only by or under the supervision of authorised personnel?					
88	Does the handling of animals by personnel appear to be consistent with the animal's welfare?					
89	If there are any animal-training programmes in place at the institution, are they documented and appropriate in technique, duration and purpose?					
90	Are appropriate barriers used to assist with training?					
91	Is physical punishment of the animals ever used?					
92	Is there regular monitoring and review of animal contact and training programmes?					
	**Transactions and Transport**	**A**	**Q**	**UA**	**Nap**	**Nass**
93	Are there facilities for crating and transporting animals?					
94	Are routine veterinary examinations performed prior to transport?					
95	Is quarantine implemented on arrival of acquisitions?					
96	Is animal population management regularly reviewed?					
97	Are acquisition and disposition activities legal and ethical?					

98	Are there records kept of the movement of animals into and out of the institution?					
	**Health & Safety**	**A**	**Q**	**UA**	**Nap**	**Nass**
99	Are there procedures and equipment in place in the event of a dangerous animal escape?					
100	Are the emergency protocols practised and if so, how often?					
101	Are records kept in the event of an animal escape/attack?					
102	Do staff receive training in animal health, disinfection principles and hygiene practices?					
103	Does the facility have clear procedures for working with hazardous animals?					
	**Other**	**A**	**Q**	**UA**	**Nap**	**Nass**
104	Are there adequate provisions for the proper storage and disposal of animal waste?					
105	Does the facility have continuing financial support?					
106	Does the total financial support appear to be adequate to meet the needs of the facility?					
	**Personnel**	**A**	**Q**	**UA**	**Nap**	**Nass**
107	Is the staff adequately directed?					
108	Is the staffing level appropriate to provide the required standards of animal husbandry and care?					
109	Do staff members regularly meet to discuss problems and possible solutions?					
110	In general do there appear to be good working relations in the zoos?					
111	Are animal care staff up to date with developments in their field of expertise?					
112	Is there provision for staff training and further development?					
